# Comparing the Blood Response to Hyperbaric Oxygen with High-Intensity Interval Training—A Crossover Study in Healthy Volunteers

**DOI:** 10.3390/antiox12122043

**Published:** 2023-11-25

**Authors:** Anders Kjellberg, Maléne E. Lindholm, Xiaowei Zheng, Lovisa Liwenborg, Kenny Alexandra Rodriguez-Wallberg, Sergiu-Bogdan Catrina, Peter Lindholm

**Affiliations:** 1 Department of Physiology and Pharmacology, Karolinska Institutet, 17177 Stockholm, Swedenpeter.lindholm@ki.se (P.L.); 2 Medical Unit Intensive Care and Thoracic Surgery, Perioperative Medicine and Intensive Care, Karolinska University Hospital, 17176 Stockholm, Sweden; 3 Department of Medicine, Cardiovascular Medicine, Stanford University, Stanford, CA 94305, USA; 4 Department of Molecular Medicine and Surgery, Karolinska Institutet, 17176 Stockholm, Sweden; 5 Department of Oncology-Pathology, Karolinska Institutet, 17176 Stockholm, Sweden; 6 Department of Reproductive Medicine, Karolinska University Hospital, 17176 Stockholm, Sweden; 7 Division of Hyperbaric Medicine, Department of Emergency Medicine, University of California San Diego, La Jolla, CA 92093, USA

**Keywords:** exercise, HIIT, ROS, oxidative stress, hyperbaric oxygen, microRNA, gene expression

## Abstract

High-intensity interval training (HIIT) and hyperbaric oxygen therapy (HBOT) induce reactive oxygen species (ROS) formation and have immunomodulatory effects. The lack of readily available biomarkers for assessing the dose–response relationship is a challenge in the clinical use of HBOT, motivating this feasibility study to evaluate the methods and variability. The overall hypothesis was that a short session of hyperbaric oxygen (HBO_2_) would have measurable effects on immune cells in the same physiological range as shown in HIIT; and that the individual response to these interventions can be monitored in venous blood and/or peripheral blood mononuclear cells (PBMCs). Ten healthy volunteers performed two interventions; a 28 min HIIT session and 28 min HBO_2_ in a crossover design. We evaluated bulk RNA sequencing data from PBMCs, with a separate analysis of mRNA and microRNA. Blood gases, peripheral venous oxygen saturation (SpvO_2_), and ROS levels were measured in peripheral venous blood. We observed an overlap in the gene expression changes in 166 genes in response to HIIT and HBO_2_, mostly involved in hypoxic or inflammatory pathways. Both interventions were followed by downregulation of several NF-κB signaling genes in response to both HBO_2_ and HIIT, while several interferon α/γ signaling genes were upregulated. Only 12 microRNA were significantly changed in HBO_2_ and 6 in HIIT, without overlap between interventions. ROS levels were elevated in blood at 30 min and 60 min compared to the baseline during HIIT, but not during/after HBO_2_. In conclusion, HBOT changed the gene expression in a number of pathways measurable in PBMC. The correlation of these changes with the dose and individual response to treatment warrants further investigation.

## 1. Introduction

HBOT has been used for almost a century for its broad anti-inflammatory and immunomodulatory effects, but the dose is delivered according to empirically set protocols extrapolated from initial treatment of decompression sickness [1]. HBOT has been proven effective in several clinical trials, where its immunomodulatory function potentially played an important role, e.g., diabetic foot ulcers [2]; soft tissue radiation injury [3]; and inflammatory bowel disease [4]. Next-generation sequencing data has provided further insights into the complex mechanisms of HBOT. In a randomized trial on patients with ulcerative colitis, multi-omic analyses show that the beneficial effects of HBOT are mediated by a combined host–pathogen response, involving a reduction in neutrophil degranulation through the *STAT3-NLRP3*-azurophilic granule pathways and a decrease in mucus-digesting bacteria, with an accompanying increase in MUC2 and epithelial HIF-1α [5,6]. Recent randomized clinical trials concluded that 40 sessions of 2.0 atmospheres absolute (ATA) HBOT enhanced physical performance in middle-aged master athletes [7] and improved cognitive function, cardiac function, and symptoms in post-COVID-19 condition [8,9]. Oxidative stress and the modulation of redox homeostasis is central in the effects of both HBOT [10] and HIIT [11]. Similarly, exercise modulates immunity in a dose-dependent manner, with large inter-individual heterogeneity, with age and sex being important factors of variance [12,13,14]. Whether modulation of immunity in HBOT is dose dependent and if there is an optimal interval and number of HBO_2_ sessions are largely unknown [15]. There is still no clinically useful method to measure individual doses and/or responses to HBOT. Treatments may vary in pressure (1.5 to 2.8 ATA), duration (60–120 min), with or without air-breaks, and number of sessions (1–60), with likely variable effects on mitochondrial ROS production and immunity, but the dose is normally not individualized [16]. A precision biomarker for dose and better insights into the immune response could improve clinical treatment allocation considerably [12].

High-intensity interval training (HIIT) induces reactive oxygen species (ROS) formation [11], and has been shown to affect hypoxia and inflammatory pathways in human peripheral blood monocytic cells (PBMCs) [17]. HIIT has become increasingly popular for its time efficiency compared to continuous aerobic exercise training (CAET), and for demonstrating similar or better effects [18,19,20]. One bout of HIIT redistributes immune cells from blood to tissues, with the effects lasting for four to six hours [21,22]. Specifically, each exercise bout improves the efficacy of tissue macrophages and promotes recirculation of neutrophils, natural killer cells, cytotoxic T cells, and immature B cells, with a corresponding increase in immunoglobulins and anti-inflammatory cytokines [13]. Changes in gene expression seem to peak between 3 and 6 h after HIIT, lasting at least 24 h [21]. ROS are extremely short-lived and most techniques for measuring ROS are non-specific and indirect [23]. Electron paramagnetic resonance spectroscopy (EPR) is generally regarded as the gold standard for measuring ROS [24]. EPR has previously been used to measure ROS levels in blood during exercise [25] but to our knowledge has not been evaluated for hyperbaric oxygen (HBO_2_).

Interestingly, many similar pathways have been reported to be altered by HBOT and intermittent hypoxia (IH), including hypoxia inducible factors 1 and 2 (HIF-1 and HIF-2) and nuclear factor kappa-light-chain-enhancer of activated B cells (NFκB), and target genes such as vascular endothelial growth factor (VEGF) and insulin-like growth factor 1 (IGF-1); a phenomenon called “the hyperoxic–hypoxic paradox” (HHP) [26]. MicroRNAs (miR) are short, non-coding RNAs, 18–25 nucleotides long, that regulate gene expression on a post-transcriptional level [27]. miR are interesting as biomarkers in many settings, including exercise, due to their stability and involvement in various biological processes [28]. We hypothesized that a short stimulus of HBO_2_ would induce measurable changes in ROS levels, venous blood gases, and gene expression in healthy volunteers. We used HIIT as a comparative intervention known to induce measurable changes in our selected variables. The aim was to evaluate the response to HBO_2_ in order to identify potential biomarkers for future studies of the dose–response relationship.

## 2. Materials and Methods

**Figure 1:**
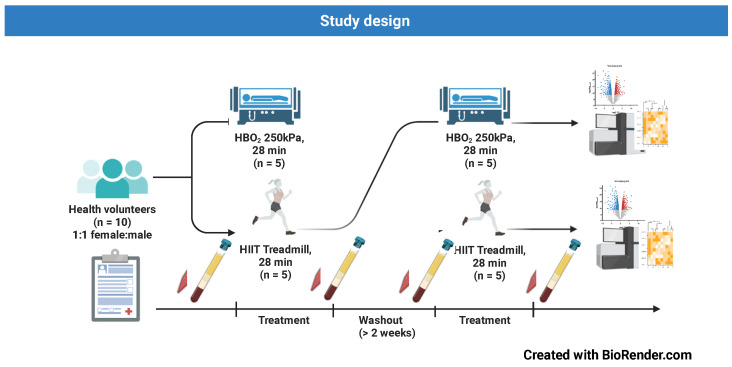
Study design.

Subjects: The study was approved by the Swedish Ethical Review Authority (EPM) (approval no. 2019-01864) and was conducted in accordance with the Declaration of Helsinki. Healthy physically active volunteers, aged 20–55, were recruited by advertisement (Table S1). After signed informed consent, 10 healthy volunteers were assigned to either HIIT or HBO_2_, depending on availability, in a crossover design with a 2-week washout period before they performed the other intervention (Figure 1). Subjects were instructed to refrain from alcohol and/or exercise for 36 h before the tests. No nicotine or caffeine and only a light snack more than one hour before the tests was allowed. The subjects ingested water as needed. Before any intervention took place, the subjects filled out a medical questionnaire and had a medical examination, including ECG, blood pressure, peripheral saturation, and chest auscultation.

Intervention protocols:

HIIT protocol: Four intervals of 3 min HIIT with a 2 min slow jog between intervals, with a 5 min warmup and a 5 min cool-down (28 min), were performed on a Skillrun™ treadmill (Technogym, Cesena, Italy). The subjects were informed about the Borg scale rate of perceived exertion (RPE) and instructed to reach equal to or above 17 (very hard) during fast intervals. The gradient was set to 1% during the warmup and slow jog and 6% during the intervals. Subjects could set their individual speed during the warmup. The interval starting speed was set by an estimation of exercise capacity according to the calculated age-dependent maximal heart rate (HR_max_), but subjects could alter the speed according to their RPE. The RPE was checked immediately after each interval (Figure S1).

HBO_2_ protocol: HBO_2_ was given in a HAUX-Starmed-Quadro 3500–2400 multiplace chamber (Haux-Life-Support GmbH, Karlsbad, Germany). Participants sat in a chair and inhaled oxygen with a tight-fitting face mask, with 5 min compression time to 2.5 ATA (254 kPa), 15 min at pressure (breathing oxygen), and 8 min decompression time (total exposure 28 min) (Figure S2). Exhaled O_2_ was measured in the hyperbaric chamber to validate that the masks were tight-fitting, for fire safety, and to make sure the dose given was the same for all subjects.

Physiological measurements: Each subject’s baseline heart rate (HR), blood pressure, including mean arterial pressure (MAP), and electrocardiogram (ECG) were monitored with a Datex-Ohmeda FM monitor (GE HealthCare, Danderyd, Sweden). The HR during HIIT was monitored using a Polar H10™ (Polar Electro Oy, Kempele, Finland) pulse monitor with a chest strap. Cadence and Watts/Calories were recorded from the Skillrun™ with the Qicraft application version 4.19.6. The respiratory rate was counted manually. HR_max_ was estimated by an online calculator provided by the Norwegian University of Science and Technology (NTNU); the HRmax Calculator is based on this formula: 211 − 0.64*age.

Blood sampling and biochemical analyses: A plastic peripheral venous catheter was inserted in the median antecubital or cephalic vein. Venous blood samples were collected at multiple timepoints: baseline, during (at 15 min for HBO_2_ and 18 min for HIIT), immediately after (30 min from start), and 60 min and 6 h from the intervention’s start; the catheter was flushed with normal saline between samples. Venous blood gas was analyzed with a ABL90 Flex plus point-of-care analyzer (Radiometer, Copenhagen, Denmark), including but not limited to pH, standard bicarbonate (_st_HCO^3−^), lactate, hemoglobin (Hb), saturation of O_2_ (SpvO_2_), partial pressure of O_2_ (pO_2_), and carbon dioxide (CO_2_).

Electron paramagnetic resonance (EPR) spectroscopy: ROS levels in the blood were measured with an EPR spectrometer (Noxygen, Elzach, Germany) using a cyclic hydroxylamine (CMH) spin probe and a CP radical standard curve. A volume of 75 µL of blood, collected in a heparin syringe, was mixed immediately with 200 µM CMH in EPR-grade Krebs HEPES buffer supplemented with 5 mM diethyldithiocarbamate (DETC) and 25 mM Deferoxamine (DFX). After incubation for 30 min at 37.5 °C, it was transferred to 1 mL syringes and snap-frozen in liquid nitrogen, then transferred and stored at −80 °C for later analysis with EPR. The spectrometer settings were as follows: microwave frequency, 9.752 GHz; modulation frequency, 86 kHz; modulation amplitude, 8.29 G; sweep width, 100.00 G; microwave power, 1.02 mW; number of scans, 15. All reagents for EPR were purchased from Noxygen.

PBMC isolation: PBMCs were isolated from blood using Ficoll-Hypaque density-gradient centrifugation CPT-tubes (BD, Stockholm, Sweden). Tubes were transferred at room temperature and centrifuged at 500× *g* for 30 min within 1 h. Citrate plasma was aliquoted and stored at −80 °C. The PBMCs were isolated, washed, and centrifuged twice with PBS buffer, and then resuspended in RNA Later™, kept at +4 °C overnight, and then stored at −80 °C until further analysis.

RNA extraction: Total RNA, including miRs, was extracted from the PBMCs with the miRNeasy Mini Kit (Qiagen, Stockholm, Sweden) as per the manufacturer’s instructions. The RNA concentration and purity was analyzed using a Nanodrop 2000 (Kodak, Stockholm, Sweden).

RNA sequencing and miR sequencing: Quality control of the extracted RNA, to check the RNA integrity and purity, was performed with an TapeStation 2200 (Agilent, Santa Clara, CA, USA). RNA sequencing was performed using single-end RNA sequencing at 150 bp length using a Hiseq 2000 (Illumina, San Diego, CA, USA) and resulted in an average read depth of 31 million reads per sample. Library preparation and sequencing was performed using the Bioinformatics and Expression Analysis Core at Karolinska Institutet. Base calling and sample demultiplexing were performed using bcl2fastq (v2.20.0), and quality and adapter trimming of reads was performed using Cutadapt (v2.8) for mRNA. For miR, adapters were trimmed with Trim Galore!, a wrapper around Cutadapt [29], an expected peak at 22 bp was detected. The sample quality was assessed using FastQC (v0.11.8). Reads were aligned to the Ensembl GRCm38 (Ensembl Homo_sapiens.GRCh38.101) reference genome and a miRNA subset of GenCode v.35 annotations, using STAR (2.7.9a). Counts for each gene were obtained using the feature Counts (v1.5.1).

Statistical analyses: Blood gas analyses were performed with the software Prism 8 (GraphPad Prism 8.4.3). A normal distribution was confirmed with the D’Agostino–Pearson and Shapiro–Wilk tests. The time courses of blood ROS, SpvO_2_, and other blood gas variables were analysed with repeated measures two-way ANOVA with Dunnett’s test for multiple comparisons. For the RNA sequencing data, the R/Bioconductor package DESeq2 [30] was used to call differential gene expression based on the gene counts generated by featureCounts. Correction for multiple testing was performed using the Benjamini–Hoschberg false discovery rate (FDR). The significance level was set to FDR < 0.05 and a log2 fold change (Log2FC) of at least ±0.5 unless otherwise stated. Principal component analysis (PCA) was performed on normalized count data. Gene ontology (GO) and gene set enrichment analysis (GSEA) were performed using the clusterProfiler package [31,32]. For GO, the PBMC gene expression data from the HBO_2_ intervention were used as the background gene set. All RNA sequencing analyses were performed using R version 4.4.2.

## 3. Results

Between 6 June 2019 and 31 October 2019, all ten participants performed both interventions; the baseline characteristics are shown in (Table 1).

**Figure 2:**
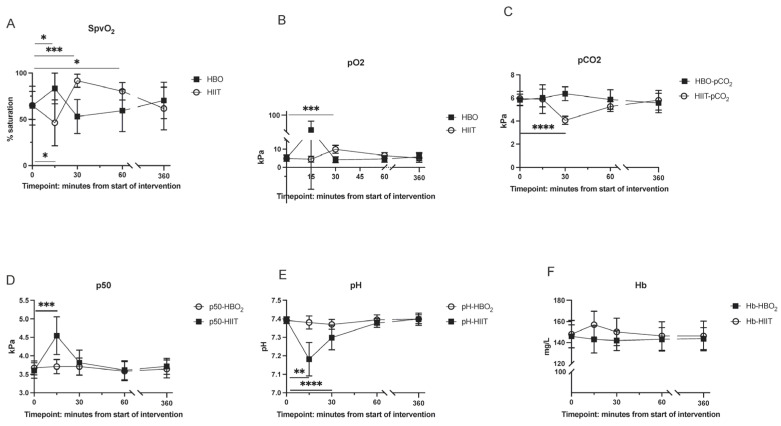
Graphs of selected blood gas data from sampled venous blood. (**A**) Peripheral vein saturation; (**B**) partial pressure of O_2_; (**C**) partial pressure of CO_2_; (**D**) calculated p50 (partial pressure at saturation 50%); (**E**) pH; (**F**) hemoglobin data (presented as mean (SD)). * indicates *p* < 0.05, ** *p* < 0.01, *** *p* < 0.001, **** *p* < 0.0001.

Physiological effects: The high-intensity exercise session was considered exhaustive (mean (SD): Borg-RPE scale, 19 (0); heart rate, 188 (5.5); 98% of estimated HR_max_). The lactate level in the blood was 14.6 (3.4) mmol/L. SpvO_2_ increased significantly during HBO_2_ but showed a tendency towards lower levels immediately after. SpvO_2_ decreased significantly during HIIT and increased immediately after, an effect that was sustained at 60 min. pCO_2_ did not change significantly during HBO_2_ but was lower at the end of HIIT (30 min). Timepoint-specific effects are shown in Table 2, Figure 2, and Table S2.

**Figure 3:**
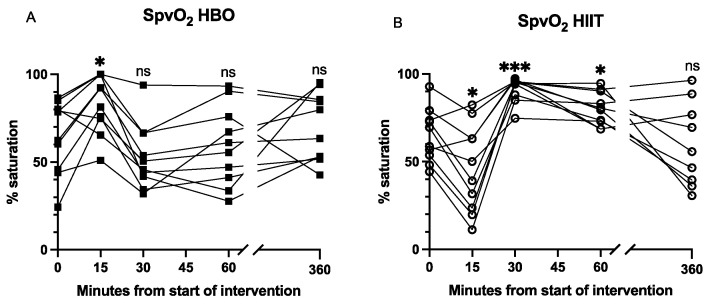
Individual changes in peripheral vein saturation. Panels (**A**,**B**) show individual values of SpvO_2_ during and after interventions. The 30 min timepoint corresponds to end of intervention. Significance level of the mean at each timepoint compared to baseline indicated by * *p* < 0.05, *** *p* < 0.001; ns, not significant.

Changes in peripheral vein saturation and partial pressure of oxygen: SpvO_2_ increased significantly during the HBO_2_ session (*p* = 0.046). There was a trend towards lower SpvO_2_ immediately after HBO_2_ (*p* = 0.20), and the level returned to baseline at 60 min. SpvO_2_ decreased significantly during the HIIT session (*p* = 0.02) but increased significantly immediately after HIIT (*p* < 0.001), remained elevated at 60 min (*p* = 0.03), and returned to baseline at 6 h (Figure 3).

**Figure 4:**
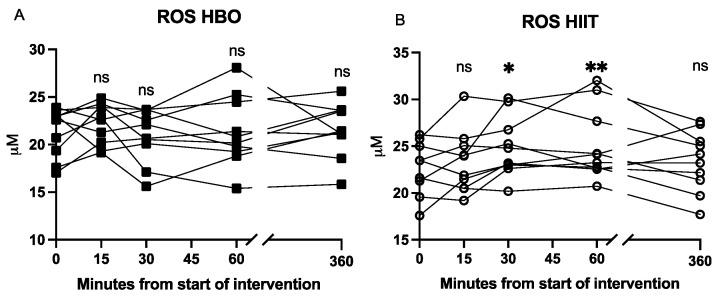
Changes in blood ROS levels. Panels (**A**,**B**) show individual values of ROS levels in venous blood during and after interventions, measured by EPR. The 30 min timepoint corresponds to end of intervention. Significance level of the mean at each timepoint compared to baseline is indicated by * *p* < 0.05, ** *p* < 0.01; ns = not significant.

ROS levels in blood: The ROS levels in the blood did not change in response to HBO_2_, whereas ROS increased from baseline at 30 min (*p* = 0.04) and stayed elevated at 60 min (*p* = 0.02) in response to HIIT. Notably, there was large inter-individual variation in the ROS levels for both HIIT and HBO_2_ (Figure 4).

**Figure 5:**
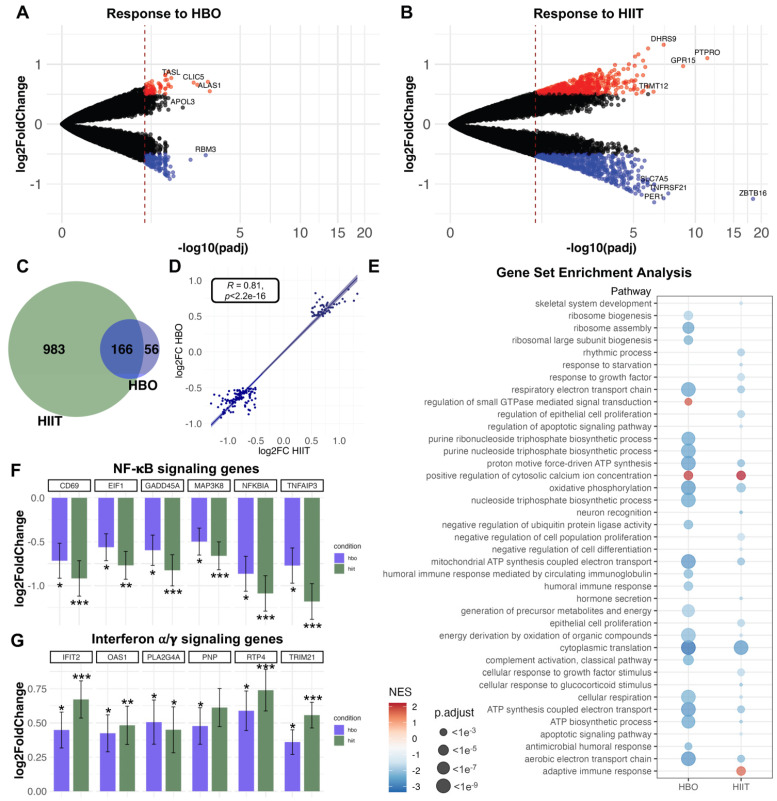
RNA sequencing results. RNA sequencing results. (**A**,**B**) Volcano plot of the log2 fold change and -log10 of the adjusted *p*-value for all expressed genes in response to HBO (**A**) and HIIT (**B**). The red dotted line indicates statistical significance (FDR < 0.05) and the colored dots indicate significant DEGs (FDR < 0.05, red for upregulated genes log2FC > 0.5, and blue for downregulated genes log2FC < −0.5). (**C**) Venn diagram of the overlap of DEGs between HIIT in green and HBO in purple. (**D**) Correlation between the log2FCs of DEGs in response to HBO and HIIT. (**E**) Top pathway enrichment results from the gene set enrichment analysis, showing the top enriched pathways for both interventions (redundant pathway names have been removed for visualization purposes). Dot color indicates normalized enrichment score (NES) and dot size the adjusted *p*-value. (**F**,**G**) Gene expression changes for (**F**) NF-κB-associated genes (selected from the Hallmark TNFA signaling via NFKB pathway), and (**G**) interferon α/γ-associated genes (selected from the Hallmark Interferon Alpha and Gamma Response pathways). Bars correspond to the mean log2FC and error bars show the standard error. Significance is indicated by * for FDR < 0.05, ** for FDR < 0.01, and *** for FDR < 0.001.

RNA sequencing of PBMC: We performed bulk RNA sequencing on the total RNA from the PBMCs before and 6 h after the start of the HBO_2_ and HIIT interventions. The first principal component of the PCA separated sex, as expected. Importantly, there was substantial intra-individual variability for some individuals, while repeated samples from others largely clustered together (Figure S3). The HBO_2_ intervention resulted in 222 differentially expressed genes (DEGs): 69 upregulated and 153 downregulated genes after 6 h compared to baseline (Figure 5A). The HIIT intervention (baseline compared to 6 h after) in the same individuals altered the expression of 1149 genes: 533 upregulated and 616 downregulated genes (Figure 5B). While the effect of HIIT on differential gene expression was more pronounced, there was a significant overlap between the genes altered in response to both HBO_2_ and HIIT (n = 166, Figure 5C). To further compare the responses between the two interventions, we correlated the log2 fold changes in the common DEGs between HIIT and HBO_2_. There was a highly significant correlation (Spearman’s rho of 0.81, *p* < 2.2 × 10^−16^) of the PBMC expression changes 6 h after HIIT and HBO_2_ (Figure 5D). Next, we performed gene ontology analysis of the up- and downregulated genes in the two interventions (using all genes with an FDR < 0.05). The HBO_2_ downregulated genes were associated with ribosomal translation, non-coding RNA processing, and apoptosis (Supplementary Figure S5A). The downregulated genes in response to HIIT were also associated with apoptosis and translational initiation, but also with cellular proliferation and growth, and response to oxidative stress (Supplementary Figure S5B). To account for all the genes without including an arbitrary significance cutoff, we performed a rank-based GSEA. The top enriched pathways in response to both HBO_2_ and HIIT are shown in Figure 5E (highly similar pathways have been removed for visualization purposes). In addition to the pathways identified through GO, we observed downregulation of several immune response pathways and mitochondrial oxidative respiration in response to HBO_2_, a positive enrichment of calcium regulation in response to both interventions, and an upregulation of the adaptive immune response in HIIT. Of particular interest, we observed downregulation of several NF-κB signaling genes in response to both interventions (Figure 5F). The NF-κB inhibitors *NFKBIA* and *TNFAIP3* were two of the most downregulated genes in response to HBO_2_. In contrast, several interferon α/γ signaling genes were upregulated in response to both HBO_2_ and HIIT.

MicroRNA (miR) in PBMCs: Further, we performed RNA sequencing of miR. The significance level was set to FDR < 0.05 and the fold change was set to 1.5 (Log2FC ±0.585) to include a few more miR for exploratory reasons. Two miR were downregulated and four upregulated in HIIT vs. four down- and eight upregulated in HBO_2_, some of them without annotated target genes. We searched the miRTarBase and GeneCards databases for associated protein coding and long non-coding genes and gene ontology. We report miRs with strong evidence for target gene association including from a reporter assay, Western blot, and/or qPCR in each intervention (Table 3).

## 4. Discussion

We reported here a clear transcriptomic response signature in PBMCs in response to a short burst of HBO_2_. Moreover, we identified common transcriptional changes in PBMCs in response to both HBO_2_ and HIIT that were associated with translational processes, cell survival, and apoptosis that might be explained by the “hyperoxic–hypoxic paradox” in immune cells. To the best of our knowledge this is the first time next-generation sequencing (NGS) has been used to compare the effects in humans of HBO_2_ and HIIT on PBMCs in vivo.

Changes in the expression of genes associated with hypoxia and inflammation were of specific interest for the hyperoxic–hypoxic paradox. Among the top 20 regulated genes in response to both conditions (Figure 5); *CD69* is an early marker of lymphocyte activation, with a complex regulatory function of the immune response, particularly in T cells and natural killer cells, and is associated with various autoimmune/chronic inflammatory diseases such as systemic sclerosis, systemic lupus erythematosus, asthma, and chronic bronchitis [33]. *CD69* regulates the differentiation of regulatory T cells and the secretion of IFN-gamma, IL-17, and IL-22. Transcription of *CD69* is detected as early as 30–60 min after stimulation but declines after 4–6 h [34]. A downregulation of *CD69* at 6 h suggests an immunomodulatory effect with a change in T-cell homeostasis [35]. *EIF1* codes for eukaryotic translation initiation factor 1 (eIF1), which plays a crucial role in the regulation of the endoplasmic reticulum (ER)/unfolded protein response (UPR). UPR is a cellular stress response pathway that is associated with many chronic inflammatory diseases, especially those related to protein misfolding, ER stress, and disrupted protein quality control such as cancer, neurodegeneration, and diabetes [36]. Inhibitors of genes in the main ER/UPR pathways are, hence, suggested as potential drug targets in these diseases [37]. Regular exercise is known to reduce ER stress with a downstream reduction in inflammation and apoptosis, and increase in nitric oxide availability, with a subsequent positive effect on endothelial dysfunction [38]. Downregulation of *EIF1,* part of the UPR, may as such either be a marker of ER stress or an adaptive effect that can explain reduced ER stress. *GADD45A*, a p53-regulated gene that codes for growth arrest and DNA damage-inducible 45a protein (Gadd45a), belongs to a group of small proteins that act as sensors of oxidative stress in many physiological processes including the UPR, with upregulation resulting in cell-cycle arrest, DNA repair, cell survival and senescence, or apoptosis [39]. Downregulation of Gadd45a suggests either a reduction in ER stress or a cellular response to ER stress that regulates UPR [40]. *MAP3K8* is a known target of HIF involved in the regulation of immune responses, including the polarization of macrophages and T-cell responses, where hypoxia upregulates MAPK expression, resulting in increased tumor necrosis factor alpha (TNFα) and other inflammatory cytokines [41]. Regulation of *MAP3K8* is complex, but a downregulation in this setting may suggest an anti-inflammatory effect [42]. *NFKBIA* codes for one of three inhibitory κB (IκB) proteins regulating NFκB. IκBα has a complex dynamic role in regulation of TNF-induced NFκB target genes [43]. *AIP3* codes for tumor necrosis factor alpha-induced protein 3, also known as A20, is a key regulator of inflammatory signaling to preserve tissue immune homeostasis, and is involved in a plethora of chronic inflammatory and auto-immune diseases [44]. Taken together, a future study is warranted to elucidate if and how the changes in gene expression related to UPR, inflammation, mitochondrial oxidative respiration, and apoptosis can be correlated with the benefits seen with different doses of HBOT.

At six hours, miR-328 was the most upregulated miR in response to HBO_2_. Its association with hypoxia regulation makes it an interesting biomarker for the dose–response relationship of HBO_2_ in health and disease [45,46,47]. From miRNA sequencing, miR-6741 was one of the most significantly upregulated miRs after HBO_2_ but was downregulated in response to HIIT (*p* < 0.011). Interestingly, miR-6741 was recently described as a potential biomarker for the severity of COVID-19, where a transient upregulation after dexamethasone treatment was associated with a poor prognosis; *APOBEC3H* and *HNRNPA1L2*, involved in antiviral defense, were identified as target genes [48]. miR-328 and miR-6741, as targets for oxidative stress, may be potential biomarkers for the HBO_2_ dose–response relationship and warrant further study. The timing of blood sampling is an important factor when assessing the effect of both HIIT and HBOT since both interventions may first induce a mild inflammatory response but later have the beneficial anti-inflammatory effects [22,26,49]. We chose six hours from start of the interventions to maximize the chance of measuring the peak of the changes in gene expression while reducing the risk of measuring the effect of the redistribution of immune cells.

EPR measurement of ROS levels was feasible for HIIT but difficult to use during HBO_2_ treatment since the venous samples taken at pressure during HBO_2_ would have to be decompressed prior to analysis, with potential influence from the sudden change in pO_2_. Future efforts to evaluate ROS levels after snap-freezing the samples in the hyperbaric setting (a procedure that could be feasible in a multiplace chamber) are needed to potentially solve this issue.

The blood gas analysis of venous samples showed a non-significant decrease in SpvO_2_ after HBO_2_. A previous study, with the hypothesis that the remaining increase in oxygen content is caused by HBOT, concluded that a single HBO_2_ treatment at 2.5 ATA for 90 min did not raise SpvO_2_. It also found a decrease in SpvO_2_ three minutes after HBO_2_, which was explained by venous stasis, although no baseline measurement was recorded [50]. We did not use venous stasis in our experiment. A transient change in pO_2_ and delta-pO_2_ would better explain the hyperoxic–hypoxic paradox and the numerous studies suggesting benefits from HBO_2_ pre-conditioning [51]. Air-breaks may be just as important in this respect. We observed a large inter-individual difference in SpvO_2_ which may reflect the redox balance in blood. This finding should be verified in a larger cohort as our sample size was limited. The pO_2_ apparatus was not validated for hyperbaric use (0–107 kPa), requiring decompression of the samples before analysis, with the resulting range 3.93–107 kPa, suggesting measurements during HBO_2_ were inaccurate. The changes in HIIT were significant and expected (Table S2), serving as a validation of the blood gas analyses.

The large individual variation seen in most analytes measured may be explained by a number of factors, including variable age, sex, food intake, and circadian effects. The results highlight the need for consideration of these important factors for HIIT and HBO_2_ when evaluating transcriptomics and other potential biomarkers of dose–response in future clinical trials. For example, we saw a significant sex difference at baseline in the transcriptomic response (Figure S3). In clinical practice of hyperbaric medicine, a “one dose fits all” approach is typically used and sex difference is not normally considered. A validated biomarker for dose–response of HBO_2_ would allow this important stratification.

Limitations: This study has some important limitations. First, the study was planned as a feasibility study to evaluate methods and logistics, and as an opportunity to test protocols that could be used in clinical trials with HBOT. The small sample size, including both sexes, reduced the power of our results. Hence, the results should be evaluated as exploratory and hypothesis generating. In particular, the changes in gene expression were based on bulk RNA sequencing of PBMCs without adjustment for differences in subsets of PBMCs. Considering the known effects of HIIT on immune cells, some of our results may reflect a cellular redistribution, despite collection of PBMCs at six hours after the start of the interventions. To gain further insights into the effects on immune function, a subset analysis and single cell sequencing should be considered in future studies.

Secondly, the HBO_2_ dose that was used is a commonly used dose for oxygen toxicity in divers (less than one third of what is normally used in clinical practice) and not intended for medical treatment.

Third, all analytes demonstrated individual variation despite the crossover design. Importantly, some of the effects may be attributed to circadian or dietary effects and the menstrual cycle in women; we cannot rule out that some of the changes seen were influenced by these factors and not solely an effect of either intervention. A standardized food protocol or overnight fasting, timing of the menstrual cycle in female subjects, and the circadian rhythm should be implemented in future studies.

## 5. Conclusions

HBO_2_ changed gene expression in a number of pathways measurable in venous blood, suggesting that PBMCs could be evaluated further in search of a biomarker for the effect of HBO_2_. The responses to HBO_2_ were measurable in similar physiological ranges as seen in response to HIIT. Individual variance, including sex, should be considered in future clinical trials of HBO_2_.

## Figures and Tables

**Table 1:** Subject baseline characteristics.

Subject	Sex	Age (years)	BMI (kg/m^2^)	HR_max_* (bpm)	Hb (g/L)	SvO_2_ (%)	HR (bpm)	MAP (mmHg)
1	M	34	23.8	187	159	56.7	73	103
2	M	50	23.0	198	162	69.5	72	88
3	F	22	22.0	197	136	92.8	71	88
4	M	28	24.0	179	146	73.6	51	92
5	M	26	21.0	190	151	58.7	92	93
6	F	32	21.0	191	141	44.3	77	71
7	F	40	19.4	190	145	79.1	79	89
8	F	27	27.0	169	134	53.7	75	94
9	M	48	24.0	186	155	72.8	70	101
10	F	35	22.0	191	128	48.1	70	78

BMI: body mass index; kg/m^2^: kilograms per square meter; HR_max_* is calculated based on the formula: 211 − 0.64∗age.

**Table 2:** Physiological, subjective, and effect changes during interventions.

Variable	HBO_2_, n = 10	HIIT, n = 10
HR_max_ (bpm)	66 (13.0)	188 (5.5)
Mean speed (km/h)	-	8.7 (0.9)
Distance (km)	-	4.0 (0.4)
Mean cadence (spm)	-	157.1 (13.0)
RPE	-	19 (0)
Effect (Watt)	-	825 (106.0)
Exhaled O_2_ max (kPa)	225.4 (0.15)	-
pH nadir	7.38 (0.0)	7.17 (0.1)
Standard bicarbonate nadir	24.1 (1.6)	14.0 (3)
SvO_2_ at 15–18 min (%)	71.0 (18.2)	27.1 (16.4)
SvO_2_ at 30 min (%)	55.0 (18.2)	90.2 (7.8)
pO_2_ at 15–18 min (kPa)	18.0 (31.7)	2.8 (1.3)
pO_2_ at 30 min (kPa)	4.46 (1.95)	9.9 (2.2)
Lactate at 15/18 min (mmol/L)	0.9 (0.4)	14.6 (3.4)
Lactate at 30 min (mM)	0.7 (0.2)	9.8 (2.5)
Hb at 15–18 min (g/L)	144 (12)	160 (13)

Values are expressed as mean and standard deviation (mean (SD)).

**Table 3:** MicroRNA changed in response to the two interventions, associated targets, and functions.

miR	Inter-Vention	Change	Target Genes	Function (GO Class)
miR-580	HBO_2_	Down	TWIST1	RISC complex
miR-1256	HBO_2_	Down	ICAM1, SELE, TRIM68	NA
miR-6806	HBO_2_	Down	NA	NA
miR-5582	HBO_2_	Down	NA	NA
miR-328	HBO_2_	Up	H2AC18, P53	Extracellular exosome, mRNA 3′-UTR binding, RISC complex
miR-4429	HBO_2_	Up	NA	NA
miR-6741	HBO_2_	Up	NA	NA
miR-4687	HBO_2_	Up	NA	Extracellular exosome
miR-513C	HBO_2_	Up	GNG13, DR1, BTG3	NA
miR-1262	HBO_2_	Up	ULK1	Negative regulation of gene expression
miR-3188	HBO_2_	Up	NA	NA
miR-1538	HBO_2_	Up	NA	NA
miR-452	HIIT	Down	HOXD10, KLF4, PPARA, NCOR2, NF1, BCL2, TFAP2C, CDKN2A, CDKN1A, TRA2B, SRSF1	RISC complex
miR-10B	HIIT	Down	HOXD10, KLF4, PPARA, NCOR2, NF1, BCL2, TFAP2C, CDKN2A, CDKN1A, TRA2B, SRSF1	Positive regulation of cell migration involved in sprouting angiogenesis, mRNA 3′-UTR binding, extracellular exosome, extracellular space
miR-1291	HIIT	Up	ERN1, ABCC1, SLC2A1	NA
miR-6851	HIIT	Up	NA	NA
miR-3618	HIIT	Up	NA	NA
miR-508	HIIT	Up	ABCB1, ZNRD1	mRNA 3′-UTR binding, miRNA-mediated gene silencing, negative regulation of NIK/NF-kappaB signaling, mRNA base-pairing translational repressor activity

## Data Availability

Source data including RNA sequencing data will be made available upon reasonable request. Metadata for the datasets generated and analyzed for this study can be found in the SciLifeLab repository, https://doi.org/10.17044/scilifelab.21792356.v1.

## Supplementary Materials

The following supporting information can be downloaded at: https://www.mdpi.com/article/10.3390/antiox12122043/s1, Figure S1: Typical HIIT profile; Figure S2: HBO_2_ profile; Figure S3: Principal component analysis (PCA); Figure S4. Gene ontology (GO) analysis; Figure S5: Differentially expressed genes miR; Table S1: Inclusion/exclusion criteria; Table S2: Results of blood gas analyses of venous samples; Table S3: MicroRNA DESeq2.
