# Supplementary material for: Comparing the Blood Response to Hyperbaric Oxygen with High-Intensity Interval Training—A Crossover Study in Healthy Volunteers
Source: Antioxidants (Basel). 2023 Nov 25;12(12):2043. doi: 10.3390/antiox12122043 (PMC10740875; doi:10.3390/antiox12122043)
Supplement: Supplementary file 1 [file antioxidants-12-02043-s001.zip › antioxidants-2699890-supplementary.pdf]

Kjellberg, et al Supplementary files

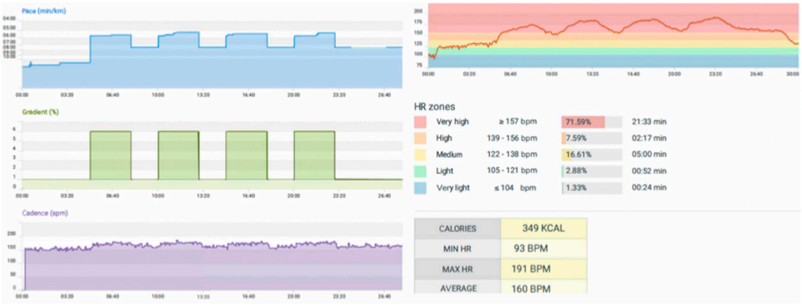

Figure S1. Typical HIIT profile showing speed, slope, cadence, time, heart rate, and estimated RPE.

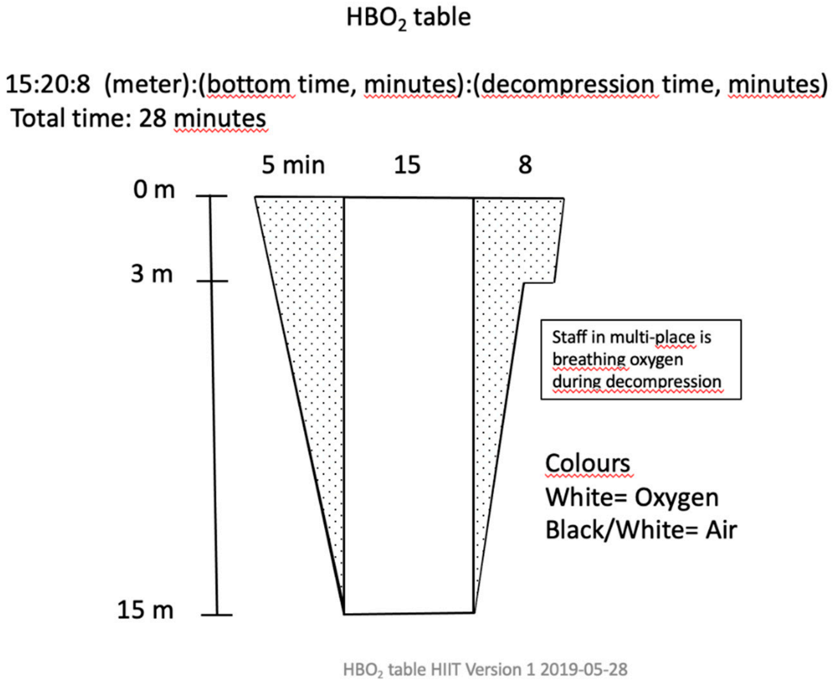

Figure S2. HBO$_2$ profile showing pressure in meters of sea water (15 m = 2.5 atmospheres absolute) and time in minutes. Dotted fields represent compression/decompression with air. White field represents subjects breathing oxygen.

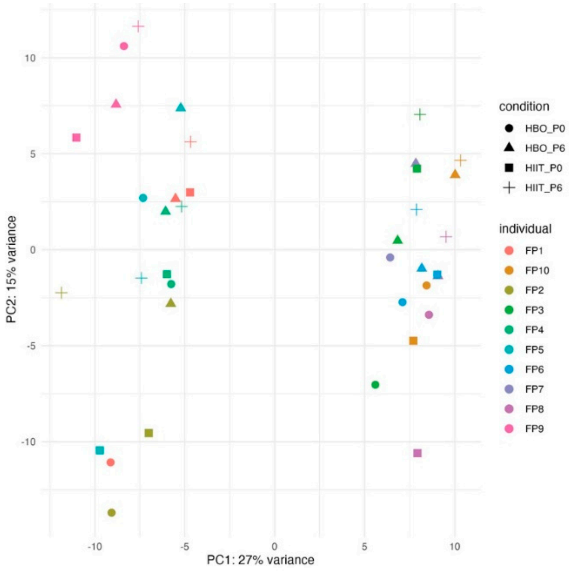

Figure S3. Principal component analysis (PCA) shows a distinct separation by sex, with 27% variance on PC1 axis with male subjects to the left and female subjects to the right. There is also a considerable individual variance, 15% on the PC2 axis.

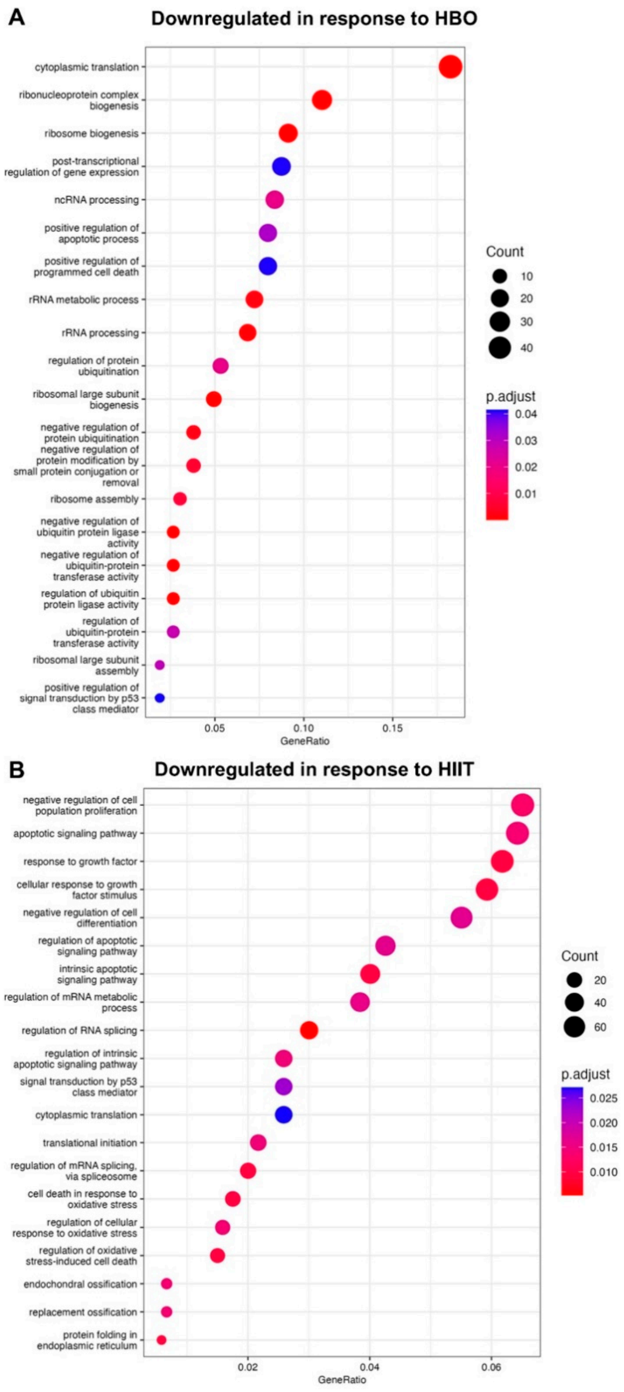

Figure S4. Gene ontology (GO) analysis, top 20 downregulated pathways in both interventions. Panel A shows pathways downregulated by HBO_2. Panel B shows pathways downregulated by HIIT. Size of the dots represents number of genes involved and color represents adjusted *p*-value.

Kjellberg, et al Supplementary files

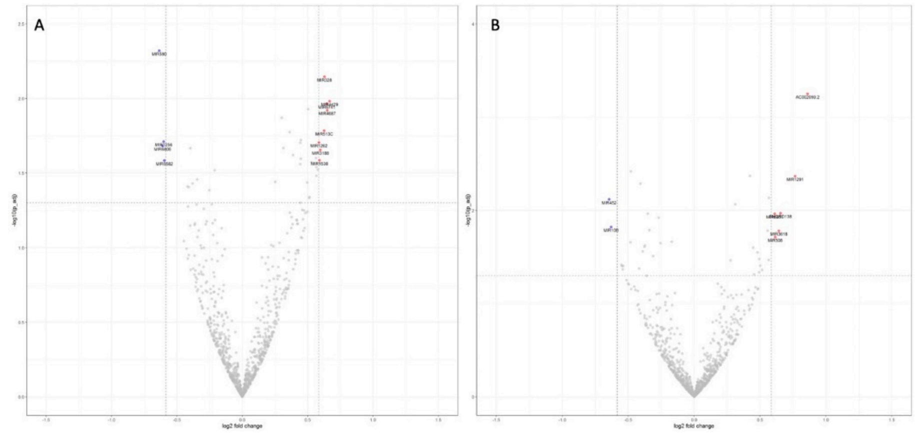

Figure S5. Differentially expressed miR genes. Volcano plot of the log2 fold change and −log10 of the *p*-value for all expressed miR genes in response to HBO_2 (A) and HIIT (B). The horizontal dotted line indicates statistical significance (FDR < 0.05) and the colored dots indicate significant DEGs (FDR < 0.05, red for upregulated genes log2FC > 0.585, and blue for downregulated genes log2FC < −0.585).

Table S1. Inclusion/exclusion criteria.

**Inclusion criteria**	**Exclusion Criteria**
Previously healthy, 20-55 years old	Smoking
Normally trained ie not elite athletes but need to cope with 15 min HIIT	Infection during the last month
Approved medical examination and health declaration according to routine for HBO treatment at Karolinska University Hospital	History of alcohol or drug abuse
Signed informed consent after both written and oral information about the study	Participants in ongoing pharmacological study
	Pregnancy
	Reluctance to participate after oral and written information
	Any severe acute or chronic medical or psychiatric condition which, according to the study leader, makes the participant unsuitable for the study

Table S2. Results of blood gas analyses of venous samples. Table shows mean (SD) for respiratory variables from venous blood gas. Statistically significant differences in change from baseline for each intervention are shown as * $p < 0.05$, ** $p < 0.01$, *** $p < 0.001$.

	**Baseline**	**Mid intervention (15/18 min)**	**End of intervention (30 min)**	**Recovery (60 min)**	**Recovery (360 min)**
pH (HBO)	7.39 (0.02)	7.38 (0.04)	7.37 (0.03)*	7.39 (0.03)	7.40 (0.02)
pO_2 (HBO_2)	5.07 (1.73)	29.97(42.13)	4.21 (1.73)	4.63 (1.89)	5.82 (2.27)
pCO_2 (HBO_2)	5.85 (0.49)	6.01 (0.77)	6.37 (0.61)*	5.87 (0.85)	5.57 (0.84)
p50 (HBO_2)	3.67 (0.19)	3.71 (0.19)	3.71 (0.23)	3.59 (0.26)	3.65 (0.24)
$SpvO_2$ (HBO_2)	64.6 (21.0)	83.3 (16.65)*	52.9 (18.5)	59.3 (22.6)	70.3 (19.7)
Hb (HBO_2)	146 (11.0)	143 (12.9)	142 (9.5)**	(143 (11.0)	144 (10.4)
pH (HIIT)	7.39 (0.02)	7.18 (0.09)***	7.30 (0.07)**	7.38 (0.02)	7.40 (0.03)
pO_2 (HIIT)	4.86 (1.52)	4.49 (1.69)	9.92 (2.21)***	6.44 (1.59)*	5.18 (2.47)
pCO_2 (HIIT)	5.95 (0.63)	5.90 (1.24)	4.06 (0.35)***	5.26 (0.43)***	5.81 (0.86)
p50 (HIIT)	3.60 (0.21)	4.55 (0.51)***	3.82 (0.34)	3.61 (0.26)	3.72 (0.21)
$SpvO_2$ (HIIT)	64.9 (15.2)	46.15(24.9)*	91.7 (7.1)***	80.3 (9.5)*	61.7 (23.1)
Hb (HIIT)	148 (13.0)	157 (12.8)***	150 (13.0)	131 (47.0)*	144 (14.6)

Table S3. MicroRNA DESeq2. Showing significantly differently expressed miR genes with Log2FC, standard error, direction of change, *p*-value, and gene name and identifier.

**Comparisons** (**Software:** DESeq2. **Method:** Wald test. Design: **~ Individual + Condition**)								
Within Treatment								
HBO2_P6_vs_HBO2_P0								
Gene name	baseMean	log2FoldChange	lfcSE	stat	pvalue	Chromosome	miRBase accession	Ensembl ID
MIR4429	2.155	0.667	0.260	2.561	0.010	2	MI0016768	ENSG00000264010
MIR4687	1.911	0.649	0.258	2.512	0.012	11	MI0017319	ENSG00000284525
MIR6741	9.781	0.646	0.253	2.547	0.011	1	MI0022586	ENSG00000284519
MIR328	410.678	0.629	0.234	2.690	0.007	16	MI0000804	ENSG00000207948
MIR513C	2.071	0.627	0.261	2.399	0.016	X	MI0006649	ENSG00000216171
MIR3188	2.995	0.597	0.261	2.288	0.022	19	MI0014232	ENSG00000267959
MIR1538	2.066	0.591	0.265	2.226	0.026	16	MI0007259	ENSG00000223109
MIR1262	8.693	0.586	0.251	2.331	0.020	1	MI0006397	ENSG00000221203
MIR580	9.976	-0.636	0.226	-2.821	0.005	5	MI0003587	ENSG00000207756
MIR6806	1.957	-0.610	0.264	-2.311	0.021	19	MI0022651	ENSG00000277588
MIR1256	7.804	-0.602	0.258	-2.336	0.019	1	MI0006390	ENSG00000221808
MIR5582	3.170	-0.596	0.268	-2.225	0.026	11	MI0019138	ENSG00000263540
HIIT_P6_vs_HIIT_P0								
Gene name	baseMean	log2FoldChange	lfcSE	stat	pvalue	Chromosome	miRBase accession	Ensembl ID
MIR1291	509.558	0.767	0.269	2.855	0.004	12	MI0006353	ENSG00000281842
MIR3618	8.705	0.643	0.269	2.396	0.017	22	MI0016008	ENSG00000284140
MIR508	10.531	0.615	0.263	2.337	0.019	X	MI0003195	ENSG00000207589
MIR6851	1.435	0.612	0.240	2.546	0.011	9	MI0022697	ENSG00000275651
MIR452	38.307	-0.645	0.242	-2.668	0.008	X	MI0001733	ENSG00000283751
MIR10B	7.811	-0.629	0.259	-2.430	0.015	2	MI0000267	ENSG00000207744

HBO2 UP

Kjellberg, et al Supplementary files

	HBO2 DOWN
	HIIT UP
	HIIT DOWN
